# Diesel Exhaust Particles Upregulate Interleukins IL-6 and IL-8 in Nasal Fibroblasts

**DOI:** 10.1371/journal.pone.0157058

**Published:** 2016-06-13

**Authors:** Jin Ah Kim, Jae Hoon Cho, Il-Ho Park, Jae-Min Shin, Seoung-Ae Lee, Heung-Man Lee

**Affiliations:** 1 Biomedical Science, Korea University, College of Medicine, Seoul, Korea; 2 Department of Otorhinolaryngology-Head and Neck Surgery, College of Medicine, Konkuk University, Seoul, Korea; 3 Department of Otorhinolaryngology-Head and Neck Surgery, Korea University, College of Medicine, Seoul, Korea; 4 Institute for Medical Devices Clinical Trial Center, Guro Hospital, Seoul, Korea; University of Rochester Medical Center, UNITED STATES

## Abstract

**Background:**

Diesel exhaust particles (DEP) are a major source of air pollution. Nasal fibroblasts are known to produce various cytokines and chemokines. The aim of this study was to evaluate DEP-induced cytokines and chemokines in nasal fibroblasts and to identify the signaling pathway involved.

**Methods:**

A cytokine and chemokine array performed after stimulation of nasal fibroblasts with DEP revealed that levels of IL-6 and IL-8 were increased most significantly among various cytokines and chemokines. RT—PCR and ELISA were used to determine the mRNA and protein expression levels of IL-6 and IL-8. Signaling pathways of p-38, Akt, and NF-κB were analyzed by western blotting, luciferase assay, and ELISA. Organ cultures of nasal interior turbinate were also developed to demonstrate the *ex vivo* effect of DEP on the expression of IL-6 and IL-8 and the associated signaling pathway.

**Results:**

DEP increased the expressions of IL-6 and IL-8 in nasal fibroblasts at mRNA and protein levels. DEP induced phosphorylation of p38, Akt, and NF-κB, whereas inhibitors of p38, Akt, and NF-κB blocked these phophorylations and the expressions of IL-6 and IL-8. These findings were also observed in *ex vivo* organ culture of nasal inferior turbinate.

**Conclusions:**

DEP induces expression of IL-6 and IL-8 via p38, Akt, and NF-κB signaling pathways in nasal fibroblasts. This finding suggests that air pollution might induce or aggravate allergic rhinitis or chronic rhinosinusitis.

## Introduction

Because of its position at the entry into the airways, the nasal mucosa is continuously exposed to inhaled agents from the environment [1]. To prevent stimulation of inflammation by these agents, the nasal mucosa has developed various mechanical mechanisms including tight junction molecules, mucus production, and ciliary movement. Moreover, it also possesses endogenous defense mechanisms involving many cytokines and chemokines [1,2]. To date, attention has mainly been paid to the mucosal role of nasal epithelium.

However, nasal fibroblasts are also known to play a very important role in various pathophysiologic conditions of the nose. Their key role is as a structural modifier of the nasal mucosa through the production of extracellular matrix [3]. Nasal fibroblasts are closely involved in mucosal hypertrophy and nasal polyps, which are common findings in allergic rhinitis and chronic rhinosinusitis [4,5]. Recent studies suggested that nasal fibroblasts are also important modulators of local inflammation by producing various cytokines, including interleukin (IL)-6 and IL-8 [6].

Diesel exhaust particles (DEP) are a key source of the particulates contributing to ambient air pollution in urban areas and can induce inflammatory responses in the upper airway. A direct causal role of DEP in allergic rhinitis or chronic rhinosinusitis has not yet been demonstrated; however, DEP is known to enhance the expression of various cytokines and chemokines in nasal epithelium. It also increases goblet cell hyperplasia and metaplastic or dysplastic change [7]. However, the effect of DEP on nasal fibroblasts has not been studied. We hypothesized that DEP might induce fibroblasts to produce various cytokines and chemokines and thus might trigger or aggravate allergic rhinitis or chronic rhinosinusitis.

The purposes of this study were to investigate the changes in various cytokines and chemokines after treatment of cultured nasal fibroblasts with DEP and to identify the underlying signaling pathways involved in the response to DEP.

## Materials and Methods

### Reagents

Inhibitors of ERK kinase (U0126), p38 (SB203580), NF-κB (BAY117082), and Akt (LY294002) were purchased from Calbiochem (Billerica, MA,USA) and were dissolved in dimethyl sulfoxide. Antibodies against phospho-ERK and phospho-p38 were purchased from Cell Signaling Technology (Danvers, MA, USA), and antibodies against phospho-Akt, NF-Kb p50 (H-119) and β-actin were purchased from Santa Cruz Biotechnology (Santa Cruz, CA, USA). Forklift-generated diesel exhaust particulates (SRM 2975, U.S. National Institute of Standards and Technology, MD, USA) were used in this study.

### Inferior turbinate tissues

To obtain inferior turbinate tissue, six patients (3 male and 3 female, mean age 35.1± 4.0) undergoing rhinoplasty were recruited. None of the patients had a history of allergy, asthma, or aspirin sensitivity and none had been treated with oral antibiotics or antihistamines for at least 2 months. Tissue was obtained at the 1cm-behind from the anterior end of the inferior turbinate under endoscopic view by using cutting forceps. Written informed consent was obtained from each patient before surgery, and the study was approved by the Korea University Medical Center Institutional Review Board (KUGH14065-001).

### Nasal fibroblast cultures

Nasal fibroblasts were isolated from tissue specimens by enzymatic digestion using collagenase (500 U/mL; Sigma), hyaluronidase (30 U/mL; Sigma), and DNAse (10 U/mL; Sigma). Cells were cultured in Dulbecco’s modified Eagle’s medium (DMEM; Invitrogen, Grand Island, NY, USA) with 10% heat-inactivated fetal bovine serum (Invitrogen, Carlsbad, CA, USA), 1% 10,000 units/mL penicillin, and 10,000 mg/L streptomycin (Invitrogen) for 4 days and then floating cells were removed by changing the medium. The purity of the nasal fibroblasts was confirmed microscopically by observing characteristic spindle-shaped cell morphology and by sorting fluorescence-activated cells. All cells used in the present study were obtained from the fourth cell passage.

### Cytotoxicity assay

To determine the cytotoxicity of DEP, the colorimetric MTT (3-[4,5dimethyl thiazol 2-yl]-2,5-diphenyl tetrazolium bromide; Sigma) assay was used. Nasal fibroblasts (4 × 10^5^ cells/mL) were seeded into 96-well tissue culture plates. After seeding, the medium was replaced with medium containing various concentrations of DEP (0–800 μg/mL). After 72 hours, the cells were incubated with MTT for 4 hours. The reaction was stopped by addition of acidified isopropanol and a microplate reader (iMark; Bio-Rad, Hercules, CA, USA) was used to measure the results at 570 nm.

### Cytokine and chemokine array

A cytokine and chemokine array (Proteome Profiler™ Human XL Cytokine Array Kit, R&D Systems, Minneapolis, MN, USA) was used to survey the changes in 102 cytokines and chemokines in nasal fibroblasts after treatment with DEP. Nasal fibroblasts (4 × 10^5^ cells/mL) from 4 cell strains were seeded into 6-well culture plates and grown until 90% confluence was achieved. After treating with or without DEP (50 μg/mL) for 72 hours, cell culture supernate was collected. It was diluted and incubated overnight with array kit. The membrane was washed to remove unbound material followed by incubation with a cocktail of biotinylated detection antibody. Strepatividin-HRP and chemiluminescent detection reagents were then applied, and a signal was produced at each capture spot corresponding to the amount of protein bound. The membrane was exposed to X-ray film for 10 minutes and profiles of mean spot pixel density were measured using Quantity One software (Bio-Rad).

### Reverse transcription—polymerase chain reaction

To evaluate the mRNA expressions of *IL-6* and *IL-8* in nasal fibroblasts after treatment with DEP, we performed reverse transcription—polymerase chain reaction. Nasal fibroblasts were exposed to various concentrations of DEP (0–50 μg/mL) for 12 hours and total RNA was isolated using Trizol reagent (Invitrogen) according to the manufacturer’s recommendations. Two micrograms of RNA were reverse transcribed using MMLV reverse transcriptase (Invitrogen). Polymerase chain reaction was performed using the following primers: *IL-6* (sense sequence 5´ GGTGCTGTCTCTCTATGCCT CTGGA 3´, antisense sequence 5´ CCCATCAGGCAAC TCGATACTCTTC 3´, 322 bp product), *IL-8* (sense sequence 5´ GGATGCTCCTGCTGTCAC 3´, antisense sequence 5´ CTGTTTGATCTGGACCTGCAG 3´, 382 base pairs). *GAPDH* (sense sequence 5´ GTG GAT ATT GTT GCC ATC AAT GAC C 3´, antisense sequence 5´ GCC CCA GCC TTC TTC ATG GTG GT 3´, 271 bp product). The gels were captured and visualized using a ChemiDoc XRS þ molecular imager (Bio-Rad).

### Quantitation of IL-6 and IL-8 by enzyme-linked immunosorbent assay (ELISA)

The protein expression levels of IL-6 and IL-8 in nasal fibroblasts after treatment with DEP were evaluated by ELISA. Nasal fibroblasts were exposed to various concentrations of DEP (0–50 μg/mL) for 48 hours and the supernatant media of cultured cells were collected. The levels of IL-6 and IL-8 were measured using ELISA (Biolegend, San Diego, CA, USA). Briefly, each well was blocked with blocking buffer for 2 hours and washed with wash buffer. Antibodies against IL-6 or IL-8 were added to the media and incubated for 2 hours. A substrate solution and stop solution were introduced sequentially and the optical density of each well was determined within 30 minutes using a microplate reader (Bio-Rad).

### Western blot analysis

To determine the signaling pathway of DEP, nasal fibroblasts were pretreated with the following signaling pathway inhibitors: LY294002 (Akt inhibitor, 10 μmol/L), SB203580 (p38 inhibitor, 10 μmol/L), or BAY117082 (NF-kB inhibitor, 2.5 μmol/L). After pretreatment, the cells were incubated with DEP (50 μg/mL) for 48 hours and then lysed in PRO-PREPTM protein extraction solution (iNtRON Biotechnology, Seongnam, Korea). Lysates were separated using 10% sodium dodecyl sulfate-polyacrylamide gel electrophoresis and transferred onto polyvinylidene fluoride membranes (Millipore, Billerica, MA, USA). Membranes were blocked using 5% skim milk solution and incubated with antibodies to phospho-Akt, p-p38, and p50, and β-actin. Blots were visualized using horseradish peroxide-conjugated secondary antibodies and an ECL system (Pierce, Rockford, IL, USA).

### Luciferase assay

Nasal fibroblasts were plated on 35-mm cell culture plates. NF-kB luciferase reporter gene constructs (luc2P/ NF-κB -RE/Hygro and hRluc/TK; Promega, Madison, WI, USA) were transfected into nasal fibroblasts using fetal bovine serum and antibiotic-free Dulbecco's Modified Eagle media containing 4 μL of purefection transfection reagent (LV750A-1, PureFection™, System Bioscience). After incubation for 20 minutes, the medium was replaced with DMEM containing 10% fetal bovine serum. After further incubation for 24 hours, the transfected nasal fibroblasts were treated with DEP (50 μg/mL). Luciferase assay was performed using a dual-luciferase reporter assay system (Promega). Firefly luciferase activity was normalized to *Renilla* luciferase activity, and the level of induction was reported.

### *Ex vivo* organ culture of inferior turbinate

Nasal inferior turbinate was cut into 2–3 mm^3^ pieces with cutting forceps. Tissue fragments were placed on a gelatin sponge (10 mm × 10 mm × 1 mm; Spongostan, Johnson & Johnson, San Angelo, TX, USA) in 6-well plates and 1.5 mL of culture medium containing Dulbecco’s Modified Eagle Medium (Invitrogen) and 2% fetal bovine serum (Invitrogen) was added to each well. The tissues were treated with DEP (50 μg/mL) with or without signaling pathway inhibitors SB203580 (10 μM), LY294002 (10 μM), or BAY117082 (1 μM) for 72 hours. The supernatant media of cultured tissues were collected to measure the level of IL-6 and IL-8 using an ELISA kit (Biolegend).

### Statistical Analysis

All the results except chemokine and cytokine array were obtained from three independent experiments using 6 different cell strains. Therefore, 18 measurements for each experimental set were collected. Cytokine and chemokine array was done 4 times using 4 different cell strains. The statistical significance of differences between control and experimental data was analyzed using unpaired *t*-test or one-way analysis of variance (ANOVA) followed by Tukey’s test (GraphPad Prism, version 5, Graph Pad Software, CA, USA). Significance was determined at the 95% confidence level and *p* values less than 0.05 were considered statistically significant.

## Results

### Cytotoxic effects of DEP on nasal fibroblasts

**Fig 1:**
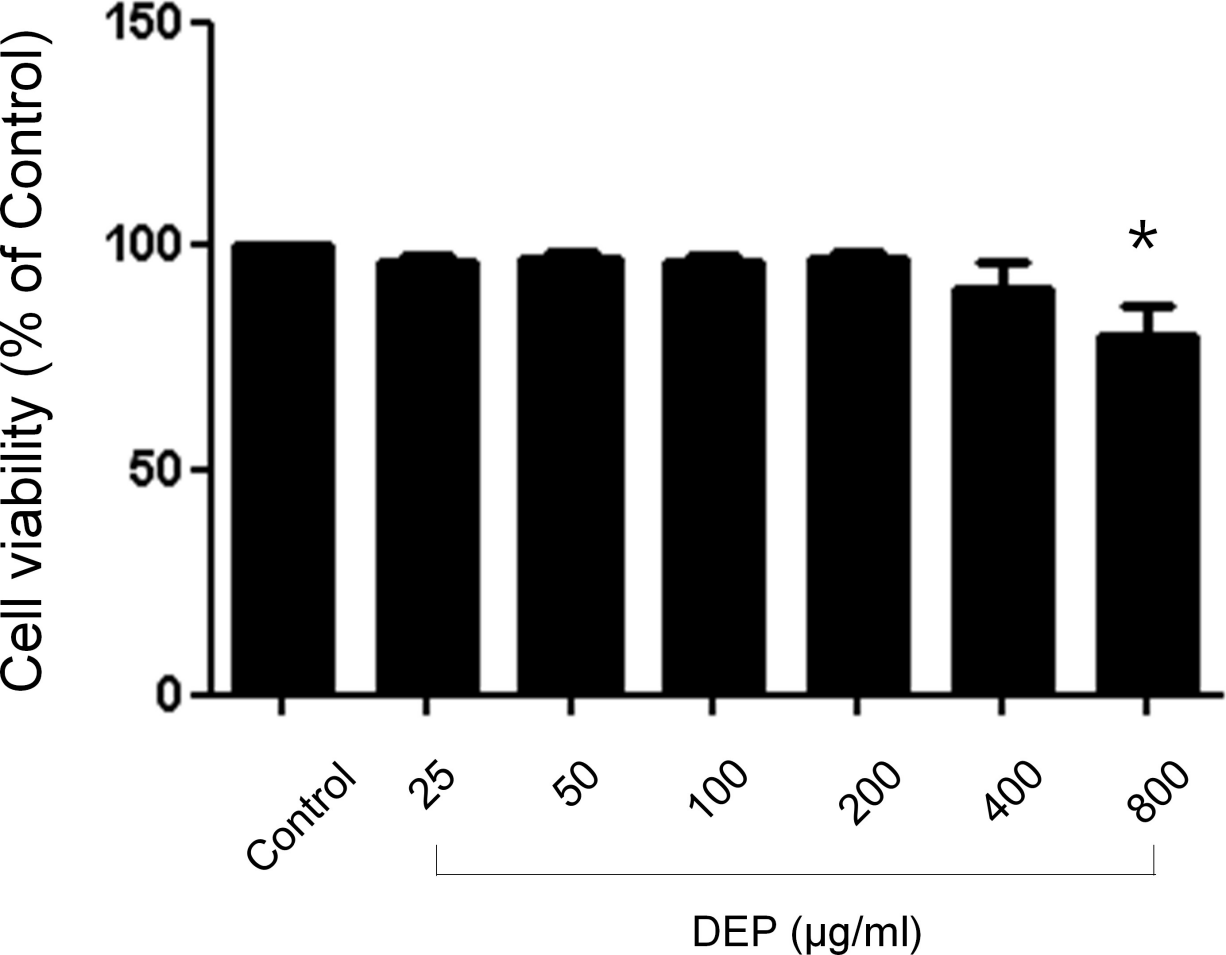
Effects of diesel exhaust particulates on viability of nasal fibroblasts. Cytotoxic effects of diesel exhaust particulates in nasal fibroblasts. Nasal fibroblasts were treated with various concentrations (0–800 μg/mL) of diesel exhaust particulates for 72 hours. Cytotoxicity tests were performed by MTT assay. DEP did not affect cell survival until the concentration reached 400 μg/mL. The graphic data represents the means ± SEM of three independent experiments. * *p*<0.05 compared to control.

To examine the cytotoxicity of DEP in nasal fibroblasts an MTT assay was performed. A cell titration curve generated from serial dilutions of nasal fibroblasts incubated with the MTT reagent indicated a linear response between cell number and absorption at 570 nm. Nasal fibroblasts were treated with DEP at concentrations that ranged from 0–800 μg/mL for 72 hours. DEP did not affect cell survival until the concentration reached 400 μg/mL (Fig 1).

### Effects of DEP on production of cytokines and chemokines in nasal fibroblasts

**Fig 2:**
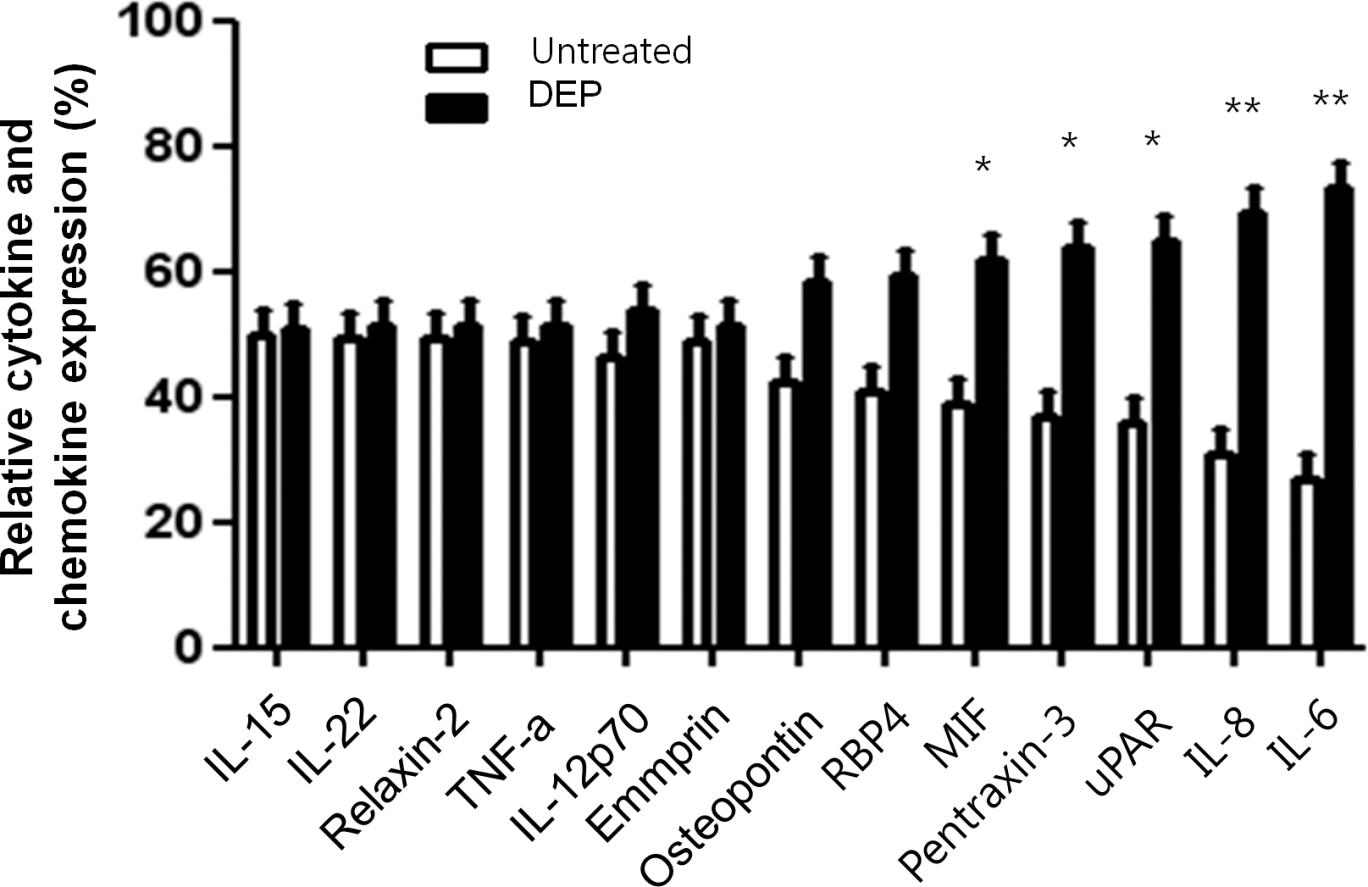
Cytokine and chemokine array in DEP-induced nasal fibroblasts. After nasal fibroblasts were untreated or treated with DEP (50 μg/ml) for 72 hours, cytokine and chemokine array was performed. Profiles of mean spot pixel density were measured using Quantity One software (Bio-Rad). The relative levels of IL-6 and IL-8 were much higher in DEP-induced nasal fibroblasts than in untreated cells. The levels of MIF, pentraxin-3, and uPAR were also higher in DEP-induced nasal fibroblasts. The graphic data represents the means ± S.E.M. for four donors. * *p*<0.05; ** *p*<0.01 compared to untreated.

After treatment with DEP (50 μg/mL) for 72 hours, DEP increased significantly IL-6 and IL-8 expressions compared to the nasal fibroblasts without DEP treatment (*p*<0.01, Fig 2). Expression of macrophage migration inhibitory factor (MIF), pentraxin-3, and uPAR also increased significantly after treatment with DEP (p<0.05). However, expression of the other 97 cytokines and chemokines among the total 102 tested were not changed and detected.

### Effects of DEP on expressions of IL-6 and IL-8 in nasal fibroblasts

**Fig 3:**
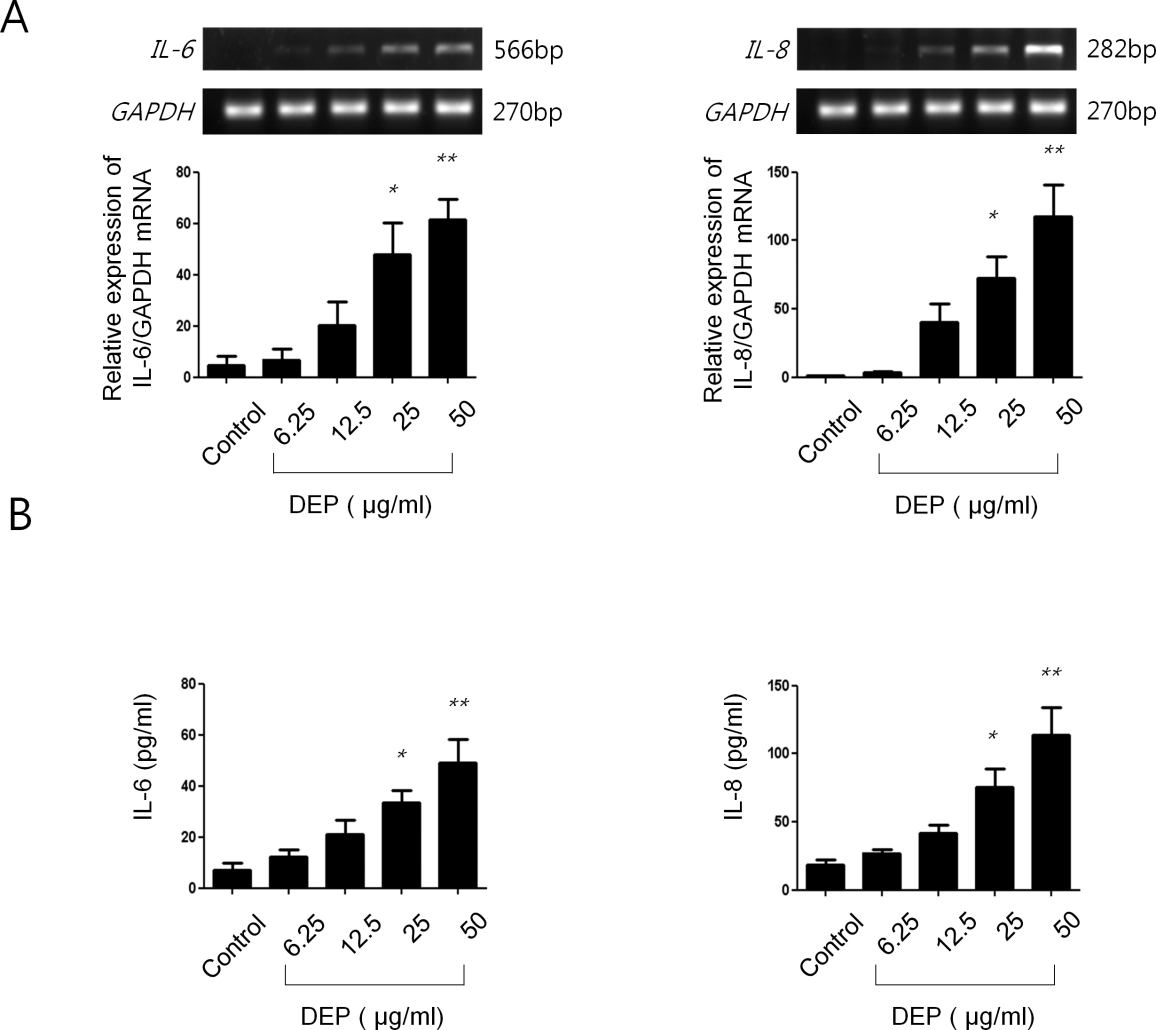
Effect of DEP on expression of IL-6 and IL-8 in nasal fibroblasts. (A) After treatment with DEP (0–50 μg/mL) for 12 hours, mRNA expression of IL-6 and IL-8 was increased in a dose-dependent manner. (B) After treatment with DEP for 48 hours, protein expression levels of IL-6 and IL-8 were also increased in a dose-dependent manner. The graphic data represents the means ± SEM of three independent experiments. * p<0.05; ** p<0.01 compared to control.

After treatment with DEP (50 μg/mL) for 12 hours, mRNA expression of *IL-6* and *IL-8* increased in a dose-dependent manner (Fig 3A). The protein expression of both IL-6 and IL-8 also increased in a dose-dependent manner after treatment with DEP (50 μg/mL) for 48 hours (Fig 3B).

### Signaling pathway of DEP-induced IL-6 and IL-8 expressions in nasal fibroblasts

**Fig 4:**
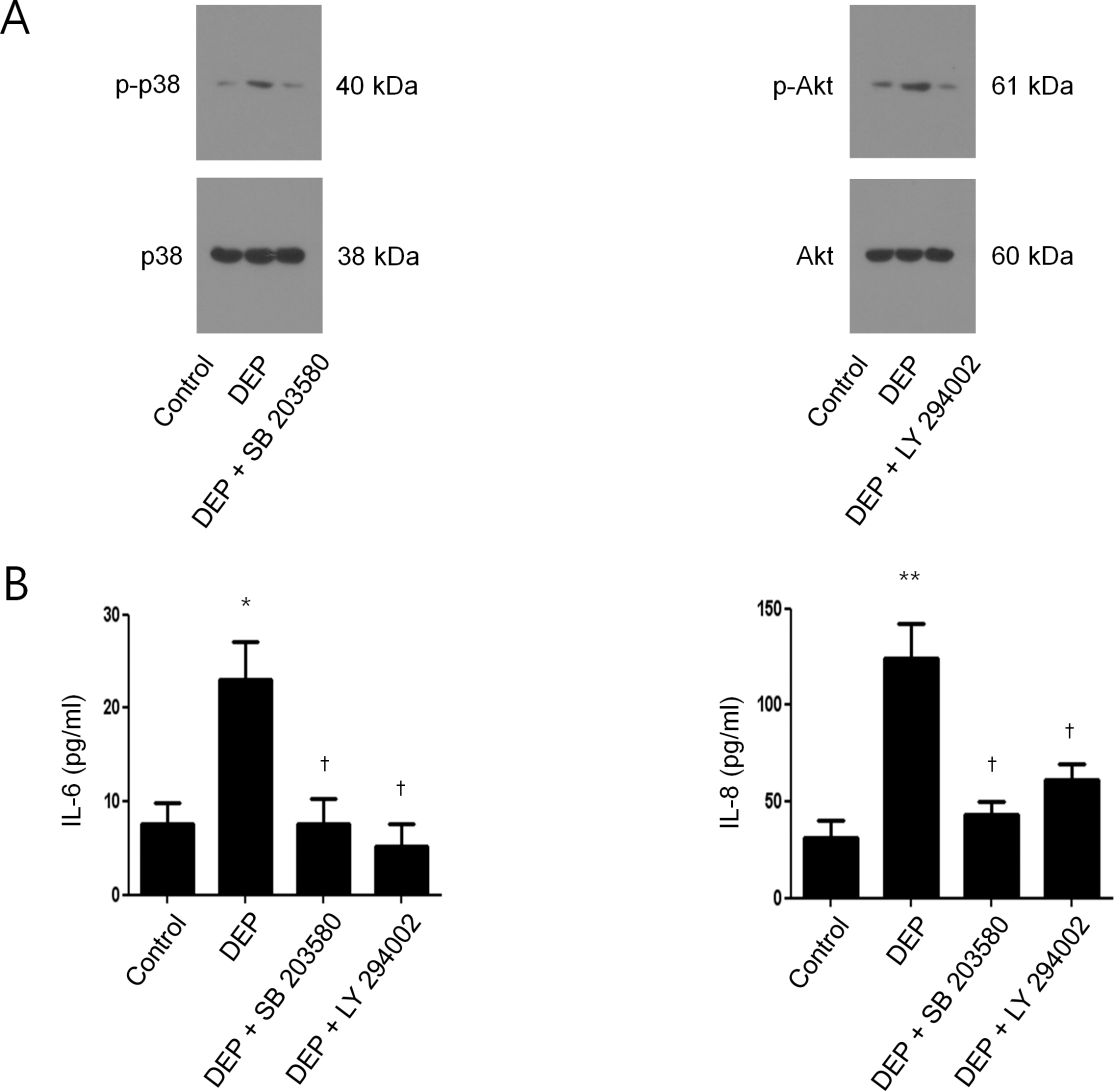
Signaling pathway of DEP-induced IL-6 and IL-8 expression in nasal fibroblasts. (A) The expression level of phosphorylated p38 (p-p38) and Akt (p-Akt) was determined by western blot in nasal fibroblast treated by DEP (50 μg/mL), in the in the presence or absence of SB203580 (p38 inhibitor, 10 μmol/L) or LY294002 (Akt inhibitor, 10 μmol/L). β-actin was used as an internal control. (B) Expression levels of IL-6 and IL-8 were measured by ELISA after treatment with DEP with or without SB203580 or LY294002. The graphic data represents the means ± SEM of three independent experiments. * p<0.05, ** p<0.01 compared to control; † p<0.05 compared to treatment with DEP alone.

To determine the signaling pathway involved in DEP-induced IL-6 and IL-8 expressions in nasal fibroblasts, we examined phosphorylated p38 (p-p38) and phosphorylated Akt (p-Akt) by western blot analysis after treatment with DEP with and without signaling pathway inhibitors (Akt inhibitor, LY294002 and p38 inhibitor, SB203580). After treatment with DEP alone, the expression of both p-p38 and p-Akt increased compared to the control. However, pretreatment with p38 inhibitor or Akt inhibitor restored the levels of expression to that of the control (Fig 4A). We also measured the level of IL-6 and IL-8 protein expression by ELISA after treatment with DEP with and without signaling pathway inhibitors. Expression of both proteins increased significantly compared to control after treatment with DEP alone but was significantly decreased by pretreatment with p38 inhibitor or Akt inhibitor (Fig 4B).

### NF-κB activation in DEP-induced nasal fibroblasts

**Fig 5:**
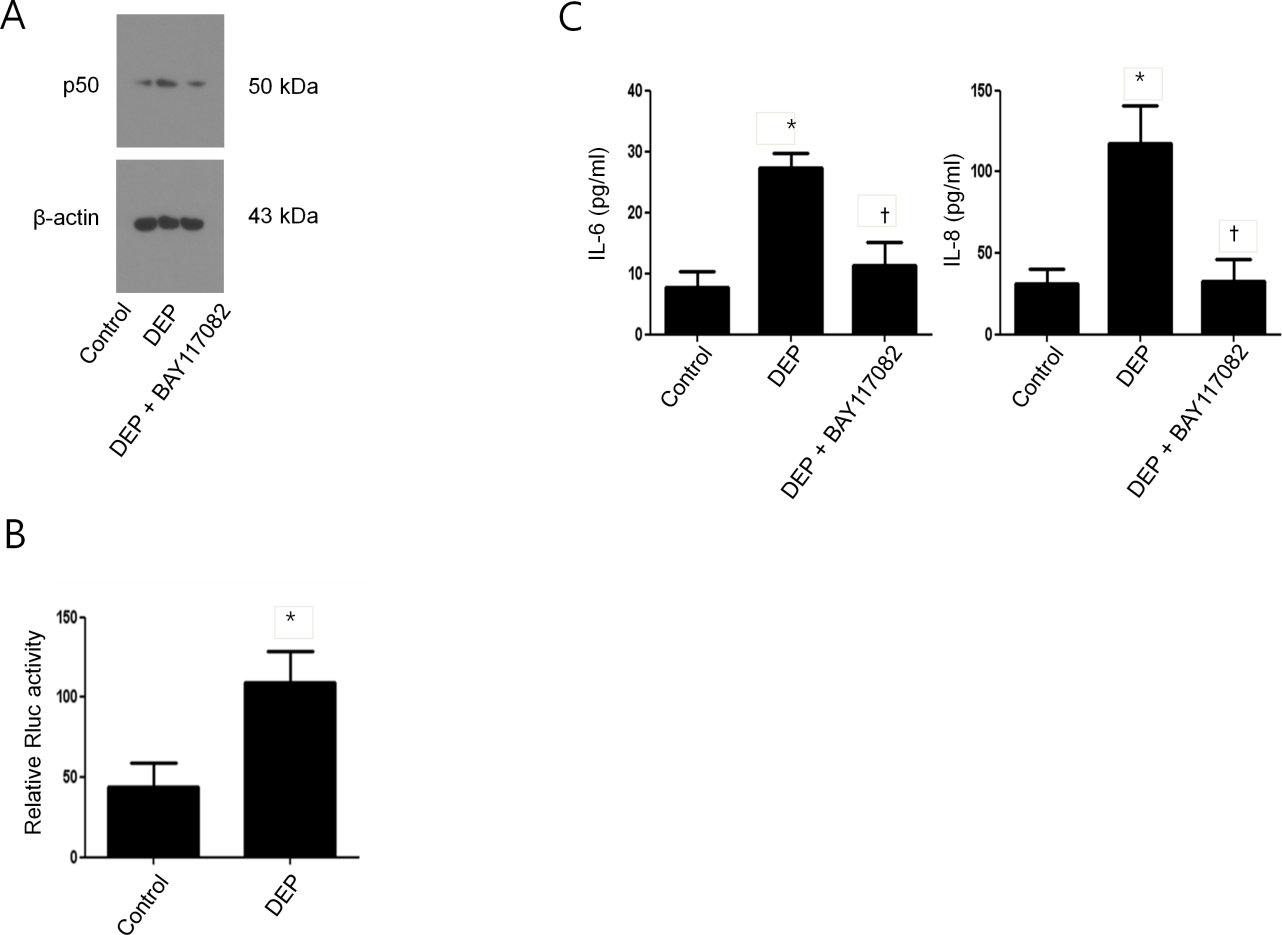
NF-κB activation in DEP-induced IL-6 and IL-8 expression in nasal fibroblasts. (A) Nasal fibroblasts were treated DEP (50 μg/mL) in the presence or absence of BAY117082 (NF-κB inhibitor, 1 μM). Protein expression levels of NF-κB p50 were examined using western blotting. (B) The transcriptional activity of NF-κB promoter by DEP was measured using luciferase assay. Firefly luciferase activity was normalized to Renilla luciferase activity, and the level of induction was reported. (C) IL-6 and IL-8 expression levels by DEP via NF-κB signal pathway were assessed by ELISA. The graphic data represents the means ± SEM of three independent experiments. * p<0.05 compared to control; † p<0.05 compared to treatment with DEP alone.

To determine the role of NF-κB in DEP-induced IL-6 and IL-8 expression in nasal fibroblasts, we examined the NF-κB subunit (p50) by western blot analysis after treatment with DEP with or without NF-κB inhibitor (BAY117082). After treatment with DEP alone the expression of p50 was significantly increased compared to control; however, this induction was suppressed by NF-κB inhibitor (Fig 5A). The transcriptional activity of NF-κB was significantly increased by DEP (Fig 5B). In addition, the NF-κB inhibitor suppressed DEP-induced expressions of IL-6 and IL-8 (Fig 5C).

### DEP-induced IL-6 and IL-8 expressions and its suppression by signaling pathway inhibitors in *ex vivo* organ culture

**Fig 6:**
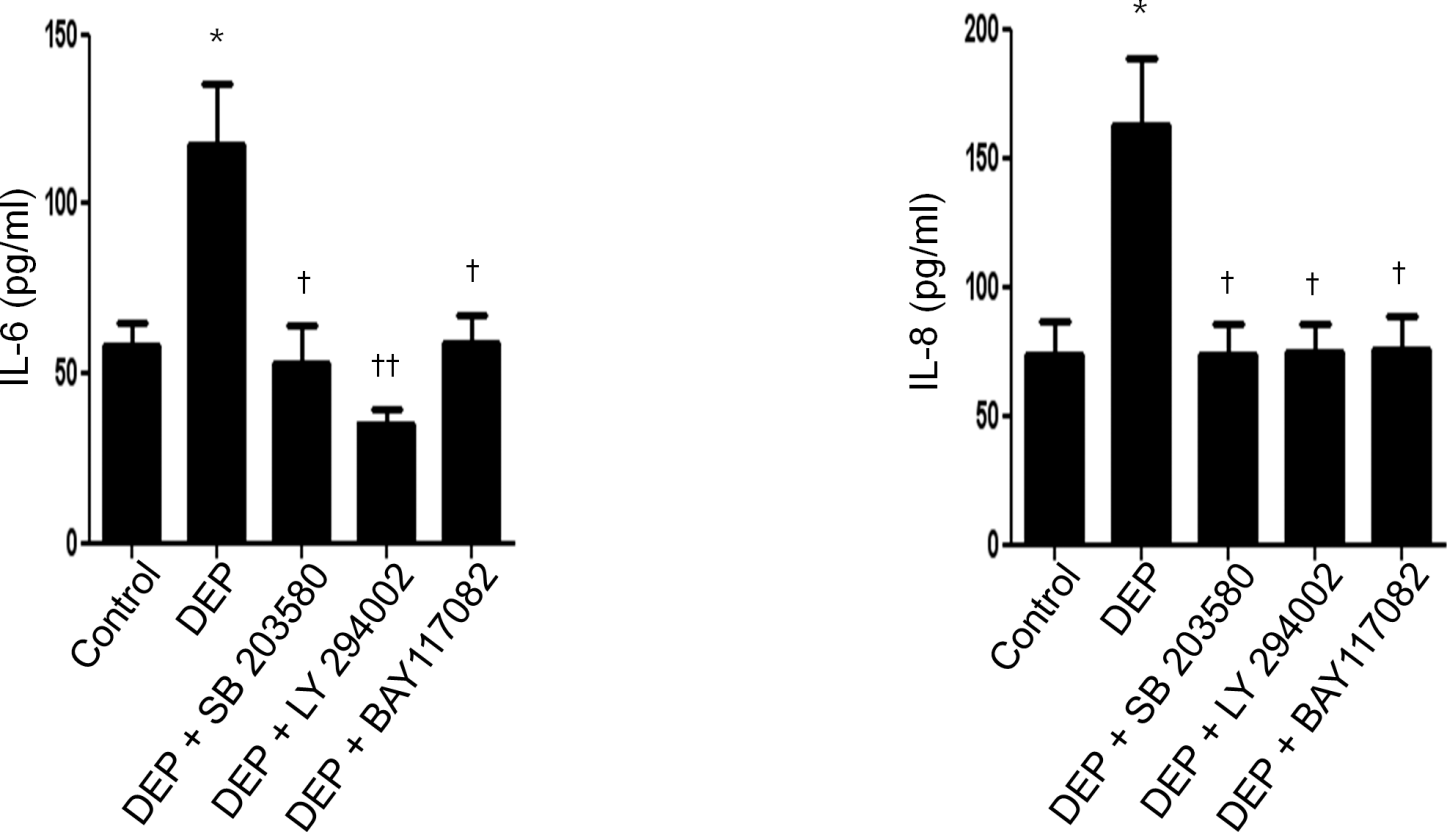
DEP-induced IL-6 and IL-8 expression and its suppression by signaling pathway inhibitors in *ex vivo* organ culture. Nasal inferior turbinate tissues were cultured ex vivo and treated with DEP (50 μg/mL). Expression of IL-6 and IL-8 was increased after treatment, as determined by ELISA. The increased expression was blocked by pretreatment with SB203580 (10 μmol/L), LY294002 (10 μmol/L), or BAY117082 (1uM). The graphic data represents the means ± SEM of three independent experiments. * p<0.05 compared to control; † p<0.05, †† p<0.01 compared to treatment with DEP alone.

To confirm the effect of DEP on the expressions of IL-6 and IL-8 in nasal inferior turbinate, we generated *ex vivo* organ cultures. The expressions of IL-6 and IL-8 were increased after treatment with DEP; however, induction of IL-6 and IL-8 was decreased by pretreatment with p38 inhibitor (SB203580), Akt inhibitor (LY294002), or NF-κB inhibitor (BAY117082) (Fig 6).

## Discussion

We can summarize the key findings of this study as follows: (1) DEP increases expressions of IL-6 and IL-8 in cultured nasal fibroblasts; (2) DEP-induced expressions of IL-6 and IL-8 is mediated simultaneously by p-38 and Akt signaling pathways; (3) DEP-induced expressions of IL-6 and IL-8 is also mediated by NF-κB; (4) all of these findings are also observed in *ex vivo* organ culture of inferior turbinate.

Air pollution adversely affects human health, especially for respiratory system. The air pollutants constitute a various particles and gases from vehicles and factories. Indoor pollutants from smoking, cooking, or burning wood in stoves and fireplace are also known to be harmful.^7^ These air pollutants are known to be risk factors for the development of asthma, bronchitis, pneumonia, and chronic obstructive pulmonary disease [8,9]. However, the effect of pollutants on upper airway disease has been minimally studied. Epidemiological studies have shown that air pollutants are also risk factors for the development of upper airway disease including allergic rhinitis and sinusitis [10,11]. Experimental work has shown that pollutants generate reactive oxygen species, induce apoptosis, and increase inflammation and mucin [12,13].

DEP is a carbon-based particulate material containing various transition metals and organic compounds [7]. Exposure to DEP is known to aggravate pre-existing allergic rhinitis. Nasal challenge of patients with allergic rhinitis showed that DEP aggravated local histamine release and symptoms and that lower allergen doses were required to trigger symptoms [14]. Combined exposure to DEP and ragweed also resulted in a strong induction of specific IgE and IgG4 in nasal lavage compared with ragweed alone. Data on pollution and rhinosinusitis are not enough. However, one study demonstrated a significant effect of raised urban air pollution levels on the prevalence of sinusitis [15].

IL-6 and IL-8 are multifunctional cytokines that are implicated in various inflammatory conditions including allergic rhinitis and chronic rhinosinusitis [16,17]. They play a key role as pro-inflammatory cytokines and modulate allergic inflammation [16]. In addition, their concentration is increased in nasal lavage and nasal polyps in chronic rhinosinusitis and they activate nasal polyp growth [6]. Fibroblasts modulate immune responses in chronic diseases by producing various cytokines or by regulating inflammatory cells [18]. We showed that the expression of MIF, pentraxin-3, uPAR, IL-6, and IL-8 was increased in DEP-induced nasal fibroblasts (Fig 2). Among these, expression of IL-6 and IL-8 was increased most significantly, and these cytokines were therefore selected for further evaluation.

Recent studies showed that DEP induces the release of IL-6 and IL-8 in various cell types [19–22]. Another study showed that DEP plays a key role in triggering an inflammatory response in skin fibroblasts [23]. However, the effect of DEP on nasal fibroblasts and the underlying signaling pathway have never been reported. Previous studies have demonstrated that DEP induces pro-inflammatory cytokines via the Akt/NF-κB pathway in mouse epidermal cells and via p38/NF-κB in bronchial epithelial cells [24,25]. On the basis of those studies, we evaluated p38 and Akt as potential downstream molecules for the signaling pathway involved in the induction of IL-6 and IL-8 by DEP in nasal fibroblasts. Our study showed increased phosphorylation of both p38 and Akt in DEP-induced nasal fibroblasts. In addition, the phosphorylation was suppressed when cells were pretreated with either p38 inhibitor or Akt inhibitor. These inhibitors also inhibited the induction of IL-6 and IL-8 by DEP. These findings demonstrated that the expression of IL-6 and IL-8 by DEP in nasal fibroblasts is mediated concurrently through both p38 and Akt.

**Fig 7:**
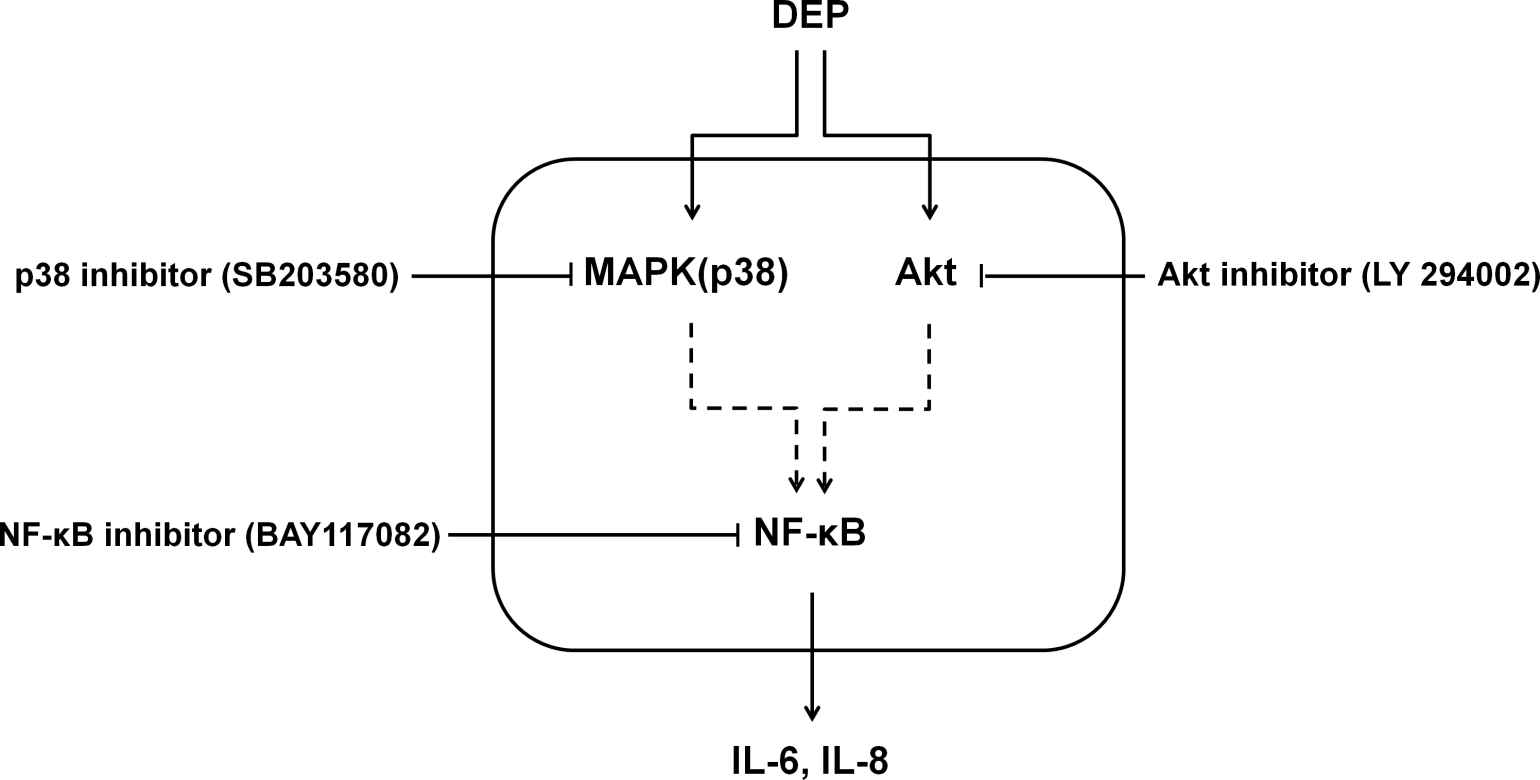
Signaling pathway for DEP-induced expression of IL-6 and IL-8 in nasal fibroblasts and organ culture of nasal inferior turbinate. DEP-induced expression of IL-6 and IL-8 is mediated by the dual signaling pathways of p38 and Akt, which converge on the NF-κB pathway.

The next step of the signaling pathway is phosphorylation of IκB, which releases NF-κB allowing it to translocate to the nucleus and activate target genes [26]. We demonstrated that NF-κB is a transcription factor in the signaling pathway of IL-6 and IL-8 induction by DEP in nasal fibroblasts. DEP increased the phosphorylation of p50, a subunit of NF-κB, and this phosphorylation was inhibited by NF-κB inhibitor. DEP-induced expression of IL-6 and IL-8 was also blocked by pretreatment with NF-κB inhibitor. Together, these findings suggest that DEP-induced expression of IL-6 and IL-8 in nasal fibroblasts is mediated by the dual signaling pathways of p38 and Akt, converging on the NF-κB pathway in common (Fig 7).

We confirmed these findings in *ex vivo* organ culture of nasal inferior turbinate tissue. The *ex vivo* organ culture system provides a real organ environment under controlled conditions [27].Nasal mucosa is composed of various cells and extracellular matrix including epithelial cells, gland cells, inflammatory cells, collagen, bone, etc. Therefore, interaction between various components might cause different result from those of fibroblasts only, so ex-vivo model was used to investigate these differences. This study demonstrated that DEP induces expression of IL-6 and IL-8 in nasal inferior turbinate tissue and that this expression is blocked by inhibitors of p38, Akt, or NF-κB.

Considering the above findings, we can infer that DEP, one of the major components of air pollution, activates nasal fibroblasts to produce IL-6 and IL-8. Increased levels of these cytokines might induce or aggravate allergic rhinitis or chronic rhinosinusitis.

## Conclusion

DEP induces expression of IL-6 and IL-8 via p38, Akt, and NF-κB signaling pathways in nasal fibroblasts, suggesting that air pollution might induce or aggravate allergic rhinitis or chronic rhinosinusitis.
